# Accelerating Progress in Women’s Sexual and Reproductive Health and Rights Decision-Making: Trends in 32 Low- and Middle-Income Countries and Future Perspectives

**DOI:** 10.9745/GHSP-D-24-00228

**Published:** 2024-12-20

**Authors:** Mengjia Liang, Lindsay Katz, Emilie Filmer-Wilson, Priscilla Idele

**Affiliations:** a United Nations Population Fund, New York, NY, USA.; b United Nations Population Fund, Toronto, Canada.

## Abstract

**Introduction:**

In 1994, the International Conference on Population and Development (ICPD) Programme of Action established the empowerment and autonomy of women as fundamental to achieving sustainable economic and social progress. Three decades later, significant strides have been made in enhancing sexual and reproductive health and rights (SRHR). However, deep-rooted gender inequality continues to impede substantial progress for many. We assess the advancements made under the Sustainable Development Goals, specifically through indicator 5.6.1, which measures women’s SRHR decision-making.

**Methods:**

Using data from 76 Demographic and Health Surveys and 1 Multiple Indicator Cluster Survey in 32 low- and middle-income countries from 2006 to 2022, the study analyzed trends in women’s SRHR decision-making and its subcomponents of autonomy on reproductive health care, contraceptive use, and sexual relations—among married or in-union women aged 15–49 years currently using contraception. The analysis also examined trends in decision-making disparities related to household wealth quintiles, women’s education levels, and area of residence, using disaggregated population-weighted percentages.

**Results:**

Analysis revealed a generally positive trend in women’s SRHR decision-making, with gains observed in Eastern and Southern Africa and notable declines in West and Central Africa. Subindicator disparities showed variations in autonomy across health care, contraception, and the ability to refuse sex. An inequality analysis highlighted that while some countries saw a narrowing wealth gap in decision-making, others faced growing disparities. Educational and urban-rural divides also shifted, reflecting a complex landscape of progress and challenges in improving women’s SRHR decision-making.

**Conclusion:**

The analysis underscores a patchwork of progress in women’s SRHR decision-making while also exposing deep disparities. These data suggest a critical need for interventions tailored to cultural and socioeconomic contexts, particularly in countries and subnational areas lagging behind. Forward-thinking strategies must prioritize enhancing women’s reproductive agency, ensuring interventions are informed by community-tailored priorities and global human rights standards.

## Abstract (precis)

This study highlights significant country and subnational variations in the progress of achieving universal sexual and reproductive health and reproductive rights, demonstrating the urgent need for targeted, culturally informed interventions to empower women globally and close persistent gaps in reproductive rights and bodily autonomy.

## Key Messages

- To assess progress in sexual and reproductive agency in the 30 years since the International Conference on Population and Development, we analyzed country-level trends in 32 low- and middle-income countries in women’s sexual and reproductive health and rights (SRHR) decision-making, specifically around Sustainable Development Goal 5.6.1, which measures women’s autonomy on reproductive health care, contraceptive use, and sexual relations.
- There were significant country and subnational variations in women’s SRHR decision-making, with notable progress in some countries contrasted by stagnation or regression in others.
- Subindicator analysis at the country level showed an increase in women’s autonomy over reproductive health care decisions and contraceptive use in more than half of the countries analyzed. However, more than half of the countries showed negative trends related to the ability to refuse sexual intercourse.
- Disparities linked to socioeconomic factors, including wealth, education, and urban-rural divides, highlight the complex interplay affecting women’s reproductive rights and bodily autonomy.
- National, regional, and global partners are encouraged to work together to ensure consistent support and funding for policies and interventions aimed at closing gaps in women’s reproductive rights and bodily autonomy.

## INTRODUCTION

The International Conference on Population and Development (ICPD) Programme of Action adopted in 1994 by 179 member states made the empowerment and autonomy of women a basis for global action to achieve sustainable economic and social progress.^1^ Now, 3 decades later, remarkable progress has been made in core areas of sexual and reproductive health (SRH). Yet, despite all that has been achieved, millions of women still see little improvement in their sexual and reproductive health and rights (SRHR) on a daily basis largely because the roots of gender discrimination run deep. The Guttmacher–Lancet Commission’s report on accelerating progress in SRHR for all emphasized a holistic approach to SRHR, integrating comprehensive service delivery with rights-based approaches.^2^ It highlighted that progress in SRHR requires addressing barriers within laws, policies, the economy, and social norms, particularly gender inequality, that hinder people’s access to SRH care.^2^ Achieving people’s well-being depends on individuals’ ability to make their own sexual and reproductive choices and respect others’ decisions.^2^ The report urged actions beyond the health sector, emphasizing reforms that promote gender equality and enhance women’s control over their bodies and lives.^2^

The United Nations Population Fund (UNFPA), with the support of an Expert Group, the United Nations Department of Economic and Social Affairs, United Nations-Women, and the World Health Organization, has successfully led efforts to develop and roll out a methodology for measuring Sustainable Development Goal (SDG) Target 5.6 of ensuring universal access to SRH and reproductive rights via SDG indicators 5.6.1—women’s ability to make decisions on reproductive health, contraceptive use, and sexual relations—and 5.6.2—number of countries with laws that enable women and men full and equal access to SRH care, information, and education—which are under UNFPA’s custodianship.^3^

These 2 indicators, introduced for the first time in the 2015 SDG framework, mark a significant step forward in addressing women’s reproductive rights—ensuring that the international development framework not only includes targets on SRH services but also targets that address women’s agency and the broader enabling legal framework for SRHR.

Indicator 5.6.1 provides new insight and has the potential to inform policy and programming interventions to boost progress toward achieving the SDGs. This indicator is only 1 of more than 200 SDG indicators to quantify decision-making by women as a matter of agency and autonomy. This differs from a past emphasis on monitoring access to services.

Through partnerships with international and regional survey programs, such as the Demographic and Health Survey (DHS), Multiple Indicator Cluster Survey (MICS), Generation Gender Survey, regional commissions, and national governments, UNFPA has increased data coverage for SDG 5.6.1 from 45 countries in 2017 to 69 countries as of February 2024.

According to data collected from 69 countries, the latest United Nations 2024 SDGs Report estimates showed that only 56% of women aged 15–49 years who were married or in a union were able to make decisions about their SRHR.^4^ These statistics showed significant disparities among regions, with data ranging from 38% in sub-Saharan Africa to more than 80% in some countries in Europe and Latin America and the Caribbean. Analysis of 3 subindicators revealed that, although 89% of women had the autonomy to decide to use contraception, about 1 in 4 women could not make their own health care decisions or say no to sex.^4^

Building on the existing evidence, we seek to capitalize on the expanded data availability for this SDG indicator to analyze trends at the country level and examine whether equal progress has been made across all groups in select low- and middle-income countries. To our knowledge, this is the first effort to examine trends in women’s decision-making in SRHR. The analysis examines the trends in SDG indicator 5.6.1 in the contexts of broader dynamics, with the understanding that these indicators are part of a broader global commitment to empower women not only by improving access to services but also by transforming the social, cultural, and legal structures that influence sexual and reproductive agency. As we assess trends over the 30-year period since the ICPD and at the midpoint of the SDGs, we aim to contextualize progress in sexual and reproductive agency within this larger framework, reflecting the evolving focus on women’s rights, autonomy, and comprehensive SRHR as foundational elements of sustainable development.

We seek to analyze trends at the country level and examine whether equal progress has been made across all groups in select low- and middle-income countries.

## METHODS

SDG indicator 5.6.1 is defined as the proportion of women aged 15–49 years (married or in union) who make their own informed decisions regarding their own reproductive health care, contraceptive use, and can say no to sexual intercourse with their husband or partner if they do not want to. A woman is considered to have autonomy in reproductive health decision-making if she (1) decides on health care for herself, (2) decides on use or non-use of contraception, and (3) can say no to sex with her husband/partner if she does not want to.

As the 2021 State of World Population report stated, “the goal of the empowerment and autonomy of women is a highly important end in itself and is essential for the achievement of sustainable development.”^5^ The SDGs take into account the ability of women and girls to make their own decisions and access health care, information, and education in reproductive and sexual matters as elemental to achieving SDG 5 on gender equality. By equipping girls and women with education, information, support, and services to determine the direction of their reproductive and sexual lives, gender inequality is reduced, public health improves, and national economies benefit.^6^

Data for this indicator are primarily sourced from the DHS. Increasingly, data also come from the MICS and other national surveys tailored to capture household demographics and health indicators. These surveys are nationally representative cross-sectional surveys of women aged 15–49 years based on a multistage stratified sampling design. Historically, the indicator has focused on married or in-union women and adolescent girls aged 15–49 who use any type of contraception. With the introduction of DHS-7 and subsequent survey rounds, as well as in other data collection instruments, including the MICS and Generation Gender Survey, the questionnaire has expanded to include all married or in-union women in this demographic, regardless of contraceptive use.

A subset of 32 countries with at least 2 surveys from different time points during the study period was used for the analysis of country-specific trends in women’s SRHR decision-making in this study. For consistency and comparability purposes, the target population in this study was limited to married or in-union women aged 15–49 years who are currently using contraception, as historical data sets predominantly captured this demographic. The data used in the trend analysis are from 76 DHS and 1 MICS. All the data used were collected between 2006 and 2022. The weighted sample sizes of women ranged from 363 in Guinea to 9,552 in Malawi. A list of countries, surveys, years, and sample sizes for the interviewed women included in this study is provided in the Supplement.

Further, for all the surveys included in the inequality analysis, the disaggregation variables and their respective levels followed the same definition. For the urban-rural analysis and wealth index analysis, we were able to use data from all 77 surveys. However, for the educational level analysis, the data from the Benin 2022 MICS survey were excluded because the disaggregation levels for women’s educational level did not align with those in all the other 76 surveys included in the analysis, meaning a direct comparison of those estimates for Benin with other countries would not be possible. Therefore, for the educational analysis, the inequality comparison made for Benin was based on the 2006 and 2018 data, while for the other 2 inequality analyses, the comparisons for Benin were based on the 2006 and 2022 data.

The primary outcome was the SDG indicator 5.6.1 on women’s SRH and reproductive rights decision-making. Given that SDG indicator 5.6.1 on women’s SRH and reproductive rights decision-making is a composite indicator comprising 3 key subindicators, we also assessed the trends for each individual component. This approach revealed variations in the subcomponents across countries, highlighting significant advancements or declines that might be obscured when only analyzing the composite indicator. To investigate the influence of sociodemographic factors on women’s SRHR, the study assessed 3 key variables: household wealth quintile, women’s level of education, and area of residence. The DHS and MICS operationalize the household wealth index by grouping households into 5 quintiles—ranging from the poorest to the richest—based on a principal component analysis. This analysis uses data on asset ownership, housing construction materials, and access to water and sanitation facilities. Women’s educational levels were categorized into 4 groups: no education, primary education, secondary education, and higher education, and women’s place of residence was classified as either an urban or rural area.^7^

We estimated the country-specific, population-weighted proportions of women who were able to make their own SRHR decisions, employing sampling weights that were calculated as the product of the inverse of the selection probability in the survey and the inverse of the response rate within each participant’s response rate group.^7^ To assess trends in prevalence from the earliest to the latest surveys, we computed country-specific estimates along with their 95% confidence intervals and standard errors. For each indicator, including each subindicator of 5.6.1 and the composite indicator, we identified the earliest and the most recent data points for each country. Using the confidence interval bounds of these points, we determined significant changes by noting whether the intervals overlapped. A lack of overlap indicated a statistically significant change from the oldest to the most recent observation. Additionally, we classified each country according to the direction of change. A positive change was noted if the most recent data point exceeded the oldest and a negative change if it was lower. We further explored within-country inequalities in adolescent motherhood across wealth quintiles, educational levels, and areas of residence. All data were analyzed in R version 4.3.3.

### Ethical Approval

All microdata files used were publicly available and fully anonymized before being analyzed. The DHS data collection procedures were reviewed and approved by the ICF International Institutional Review Board.^8^ Each DHS or MICS survey was approved by the relevant country-specific ethical review board.^8^^,^^9^

## RESULTS

### Trends and Regional Disparities

Of 32 countries with more than 1 data point investigated, levels of women’s SRHR decision-making varied greatly across countries and regions, with the lowest level in Senegal (4.9%) and the highest level in the Philippines (84.5%). West and Central Africa is home to the only 3 countries where this indicator value was less than 10%—Senegal, Mali, and Niger.

A trend analysis between the oldest and newest data points for each country, from the periods 2006–2017 and 2012–2022, indicated an overall positive trend in 19 countries. The positive changes varied, with the smallest increase being 0.9 percentage points in Madagascar and the largest being 14.3 percentage points in Uganda. In contrast, 13 countries experienced declines in women’s reproductive autonomy, with negative trends ranging from a decrease of 17.1 percentage points in Gambia to 0.9 percentage points in Nigeria.

A trend analysis indicated an overall positive trend in 19 countries, while 13 countries experienced declines in women’s reproductive autonomy.

**FIGURE 1:**
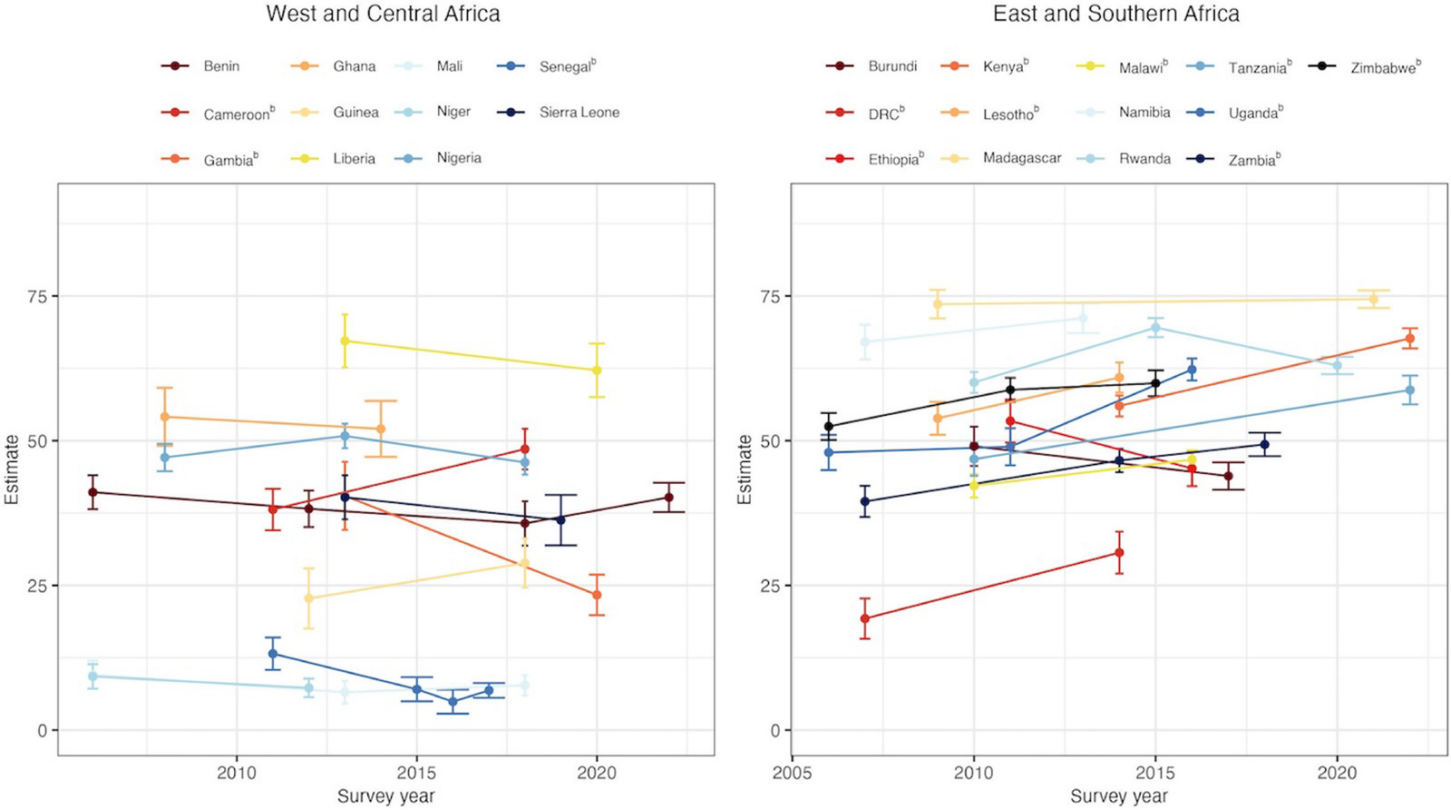
Trends in the Proportion of Women Aged 15–49 Years Who Make Their Own Decisions Regarding SRHR,^a^ West and Central Africa and East and Southern Africa, 2006–2022 Abbreviations: DRC, Democratic Republic of the Congo; SRHR, sexual and reproductive health and rights. ^a^Including deciding on their own reproductive health care, deciding on the use of contraception, and can say no to sex. ^b^Indicates a statistically significant change. Source: United Nations Population Fund, global databases, 2024.

At the regional level, the most substantial positive shifts were seen in Eastern and Southern Africa, where 11 of 13 countries showed upward trends. Seven countries reported increases of at least 7 percentage points in this indicator: Uganda (+14.3 percentage points), Tanzania (+11.9 percentage points), Kenya (+11.7 percentage points), Democratic Republic of the Congo (+11.4 percentage points), Zambia (+9.8 percentage points), Zimbabwe (+7.5 percentage points), and Lesotho (+7.1 percentage points) (Figure 1). Conversely, the most significant downward trends were observed in West and Central Africa, with 9 countries showing negative changes: Gambia (−17.1 percentage points), Senegal (−6.3 percentage points), Liberia (−5.1 percentage points), Sierra Leone (−3.9 percentage points), Ghana (−2.1 percentage points), Niger (−2.0 percentage points), Mali (−1.8 percentage points), Benin (−0.9 percentage points), and Nigeria (−0.9 percentage points) (Figure 1).

While further research is required to understand the factors behind the observed declines, the knowledge of the determining factors and variables for women’s decision-making on SRHR suggests the need to engage beyond access to quality services.^10^ The exclusion of girls from SRH education services; the strong decision-making power of family members, including husbands, over contraceptive use; and the lack of agency and empowerment of women are pushing them further behind in exercising their reproductive autonomy.

The trend analysis was limited in Arab States and Latin America and the Caribbean, with only 1 country included from each region. Similarly, in Eastern Europe and Central Asia, as well as the Asia and Pacific, data were available for just 3 countries in each. The scarcity of data in these 4 regions precluded the drawing of definitive trend conclusions at the regional level.

### Diverging Subindicators Within Countries

An analysis of the levels in the 3 subindicators revealed notable disparities across countries. For example, in Mali, the 2018 DHS survey showed a stark contrast between women’s decision-making on contraceptive use (76.7%) and health care-seeking (22.0%). In Gambia, 2020 survey data revealed that only 51.1% of the women reported being able to say no to sexual relations, whereas 85.8% of the women reported independent or joint decision-making on contraceptive use.

When assessing trends, women’s health care decision-making showed the most positive picture, with 22 of 32 countries reporting an increase in women’s autonomy over health care decisions. Notably, 15 of these improvements were statistically significant. The 5 countries that exhibited the most improvement were Haiti (13.9 percentage points), Tanzania (14.5 percentage points), Guinea (14.6 percentage points), Zambia (15.0 percentage points), and Cameroon (17.7 percentage points). Of the 10 countries that exhibited a decrease in this component, 6 are in West and Central Africa, with the top 3 largest observed decreases in Gambia (−21.6 percentage points), Niger (−9.1 percentage points), and Sierra Leone (−9.1 percentage points).

In contrast, the component related to the ability to refuse sexual intercourse exhibited the most negative trends. More than half of the countries (20 of 32) reported a decrease, particularly in West and Central Africa, where 9 of these 20 countries are located. Among these, 16 countries showed statistically significant declines. Overall, there were large variations between countries, ranging from a 16.3 percentage point decrease in Senegal to a 22.7 percentage point increase in the Democratic Republic of the Congo. Improvements in women’s autonomy to say no to sex were most visible across Eastern and Southern Africa but also Niger (+3.9 percentage points), Guinea (+4.4 percentage points), Philippines (+5.9 percentage points), and Albania (+9.8 percentage points).

Positive trends in women’s decision-making on contraceptives were observed in 19 of 32 countries, with 9 showing statistically significant improvements. A significant number of these positive changes occurred in East and Southern Africa. Thirteen countries experienced declines, notably in West and Central Africa. Benin recorded the largest decrease (-8.6 percentage points), whereas Zambia saw the most significant increase (9.1 percentage points).

The trends for the 3 subindicators sometimes diverged within a single country. For example, Benin saw an improvement in women’s health care decision-making but a decline in their ability to refuse sex and make contraceptive decisions. Such diverging trends, observed in many countries, underscore the complexity of monitoring women’s SRHR decision-making. They indicate that composite percentages may mask underlying disparities at the component level and that interventions can have varied impacts on different aspects of SRHR. This nuanced picture emphasizes the critical need to understand the underlying drivers of these trends.

### Leaving No One Behind

**FIGURE 2:**
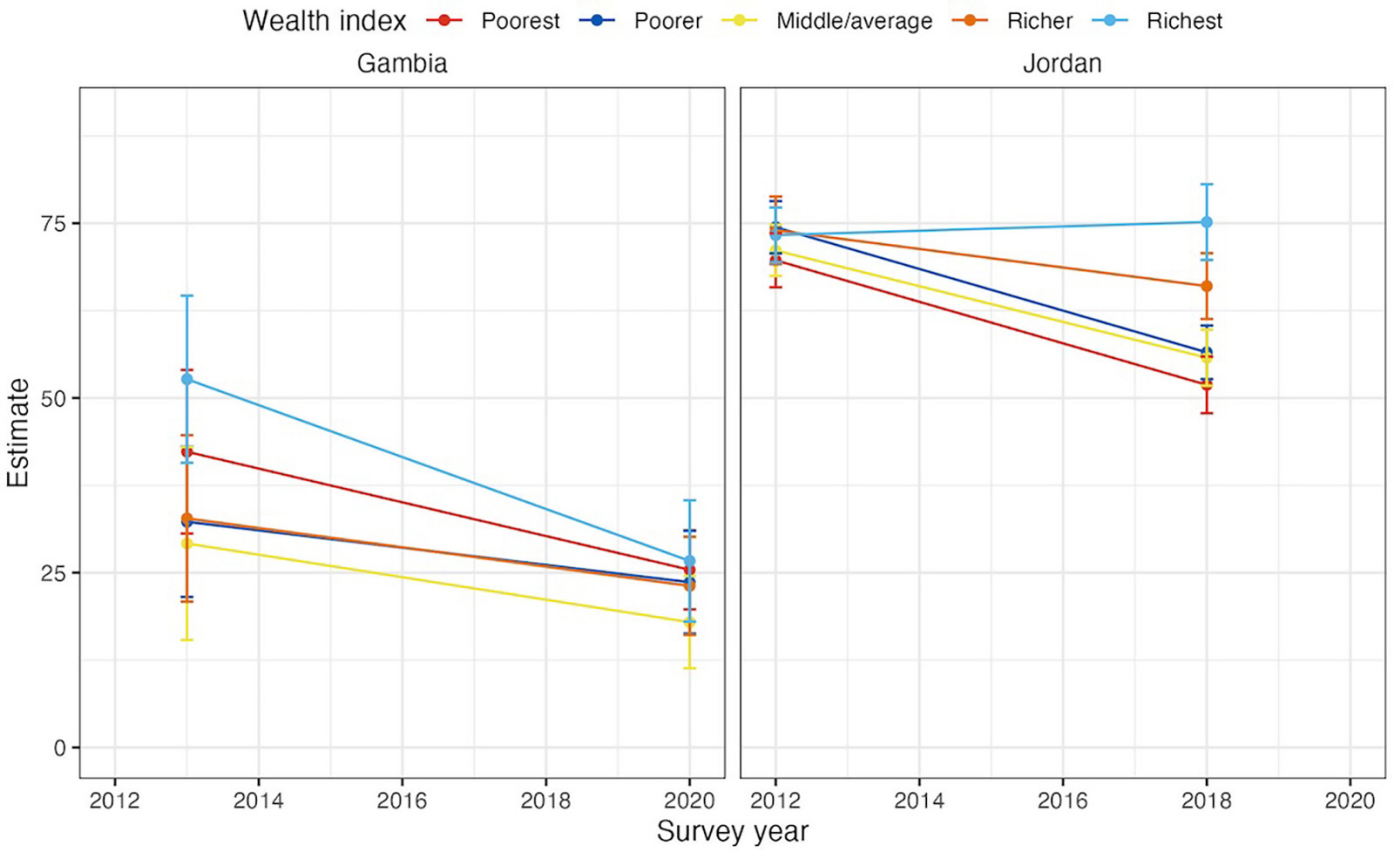
Trends in Household Wealth Disparities in the Proportion of Women Aged 15–49 Years Who Make Their Own Decisions Regarding SRHR, Gambia and Jordan, 2012–2020 Abbreviation: SRHR, sexual and reproductive health and rights. Source: United Nations Population Fund, global databases, 2024.

Inequality analysis regarding women’s autonomy in SRHR decision-making by household wealth index showed that the gap between the richest and poorest group decreased by at least 9 percentage points in 4 countries: Rwanda (−11.1 percentage points), Senegal (−10.8 percentage points), Guinea (−9.8 percentage points), and Gambia (−9.1 percentage points). In contrast, several countries exhibited an increase in the gap between the richest and poorest groups over time. The countries with the largest disparity increase include Armenia (22.1 percentage points), Cameroon (19.9 percentage points), Jordan (19.7 percentage points), Uganda (14.1 percentage points), and Tanzania (13.6 percentage points). Figure 2 provides an example of a country that exhibited a widening wealth disparity over time (Jordan) and a country that exhibited a narrowing wealth disparity over time (Gambia).

**FIGURE 3:**
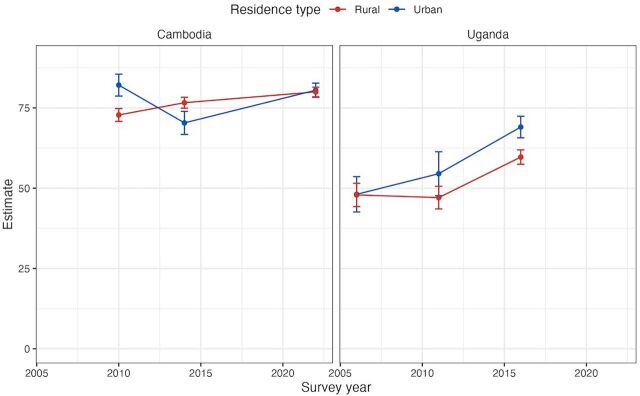
Trends in Urban-Rural Divide in the Proportion of Women Aged 15–49 Years Who Make Their Own Decisions Regarding SRHR, Cambodia and Uganda, 2006–2022 Abbreviation: SRHR, sexual and reproductive health and rights. Source: United Nations Population Fund, global databases, 2024.

The analysis further delved into trend analysis in the urban-rural divide. A decrease in the urban-rural gap was noted in several countries. Cambodia, for example, saw this disparity shrink by 8.8 percentage points from the earliest to the latest data (Figure 3). In contrast, the gap expanded in some regions. Notably, Armenia and Uganda experienced increases of 12.3 and 9.2 percentage points, respectively, highlighting a growing divide in women’s reproductive agency between urban and rural populations.

**FIGURE 4:**
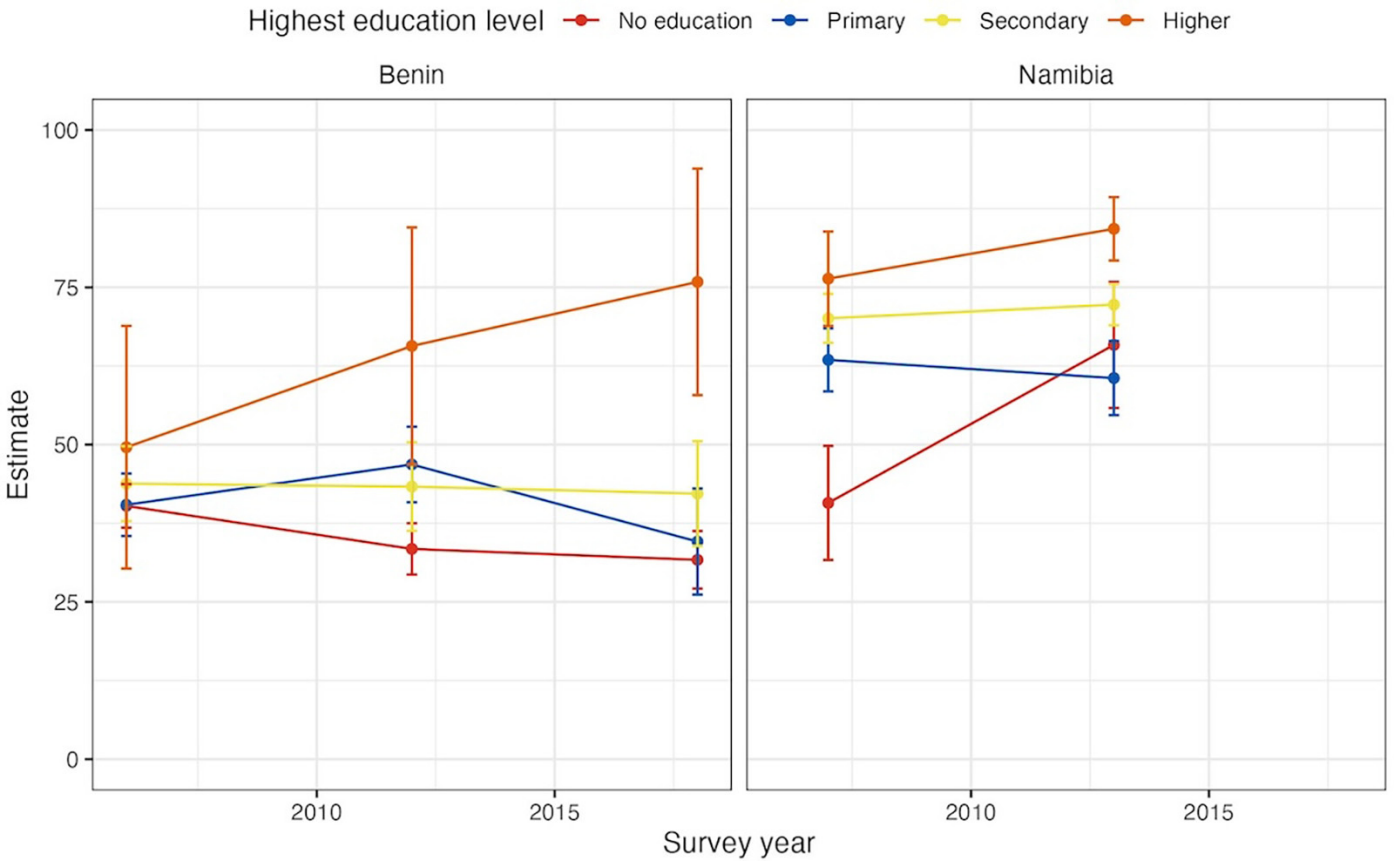
Trends in Education Disparities in the Proportion of Women Aged 15–49 Years Who Make Their Own Decisions Regarding SRHR, Benin and Namibia, 2006–2018 Abbreviation: SRHR, sexual and reproductive health and rights. Source: United Nations Population Fund, global databases, 2024.

The analysis also examined the level of education, specifically tracking changes over time between women with no education and those with higher education. This examination revealed significant shifts in the educational disparity in women’s reproductive decision-making across countries, particularly within the West and Central Africa and East and Southern Africa regions. Notably, several countries observed a reduction in the educational gap that exceeded −9 percentage points. These included Senegal, where the gap narrowed by −32 percentage points, followed by Namibia (-17.2 percentage points), Mali (−13.6 percentage points), Guinea (−11.7 percentage points), Uganda (−11.3 percentage points), and Rwanda (−9.4 percentage points), showcasing substantial progress toward closing the educational divide in these countries. However, other countries within the same regions witnessed an expansion in this educational gap. For example, Benin and Tanzania experienced a significant increase in the disparity, with the gap widening by 34.9 and 41.6 percentage points, respectively. Figure 4 includes a visualization of these trends for 2 countries, 1 in which the disparity for this indicator across educational groups grew over time (Benin) and 1 in which that narrowed (Namibia).

It is worth noting that any observed reductions in disparities must be carefully interpreted within the broader context of overall trends in women’s SRHR decision-making. In some cases, the narrowing of disparities occurred alongside declines in overall women’s SRHR decision-making, indicating that reduced inequality may reflect a regression in rights for all groups rather than equitable progress.

The analysis also examined the level of education, revealing significant shifts in the educational disparity in women’s reproductive decision-making across countries.

## DISCUSSION

The comprehensive analysis of trends in women’s SRHR decision-making across 32 low- and middle-income countries reveals a complex landscape. While there is an overall positive trend in 19 countries, 13 countries have seen declines, underscoring that progress is not universally positive. The most significant improvements were observed in Eastern and Southern Africa, yet West and Central Africa experienced notable declines, highlighting regional disparities.

This nuanced picture emphasizes the critical need to understand the underlying drivers of these trends and the context-specific nature of these drivers. Socioeconomic factors, such as household wealth, education levels, place of residence, and age of first marriage, may play a role in shaping these outcomes. For example, the analysis revealed shifts in the urban-rural gap, with some countries seeing a decrease in disparities while others, like Armenia and Uganda, displayed widening gaps. Similarly, the educational divide in countries like Namibia showed signs of narrowing, contrasting with the growing disparities in Benin and Tanzania. While multisectoral strategies that tackle the barriers that play out at the individual, interpersonal, community, and institutional levels are critical, community outreach strategies, male engagement, information technologies for development, and formative research at the initial stages of programming to understand context show promising results.^11^ Engaging men in community mobilization activities and informal education has been linked to better reproductive health outcomes. Efforts to improve dialogue between couples and explore mutually supportive gender roles is another promising approach to increase joint decision-making and introduce communication skills that improve understanding and empathy. In the area of health system strengthening, effective interventions include training health service providers on quality of care and gaps in competency, supervision of health facilities in partnership with local government health officials, and provision of contraceptives and other essential reproductive health supplies.

What affects women’s ability to decide? Both quantitative and qualitative sources, including case studies and systematic literature reviews, have identified several significant demographic and social factors influencing all areas of SDG indicator 5.6.1.^10^ These include women’s education level, her husband’s/partner’s education level, household wealth status, urban residency, and access to radio and television.^11^ SRHR knowledge is a key predictor of women’s decision-making ability. Without access to quality information, communicated effectively, it would be difficult to determine whether women actually make informed decisions. The role of the husband or partner in the decision-making process is pivotal. Men’s support can facilitate access, but usage as “joint decisions” are likely to include a substantial percentage of decisions in which women were overruled by men. The extended family, especially the mother-in-law in rural contexts, may also influence women’s SRHR decision-making processes. Communication between partners or spouses is a positive predictor of joint or women’s decision-making. Gender and cultural norms exert a profound influence. When these norms prescribe women to be submissive and passive in sexual relations, fulfill reproductive obligations, and obey their husbands’ decisions, they negatively impact women’s bodily autonomy. Issues of access, affordability, and acceptability of health services also affect decision-making. Barriers, such as disrespectful treatment by health workers, informal costs, stock-outs, and, sometimes, the absence of female health care providers, play a significant role in the decision-making process.^10^

The most significant demographic and social determinants across all areas of indicator 5.6.1 that were found in this same study were women’s education level, her husband’s/partner’s education level, household wealth status, urban residency, and access to radio and television.^11^ At an individual level, women’s knowledge of SRHR, agency, and autonomy were the most influential determinants. Strengthened support at policy and programming levels for these variables, together with an understanding of these key determinants, can motivate significant improvements in women’s SRHR decision-making.

Strengthened support at policy and programming levels for these variables, together with an understanding of these key determinants, can motivate significant improvements in women’s SRHR decision-making.

To the best of our knowledge, there have been no internationally agreed-upon best practices and interventions to strengthen women’s SRHR decision-making. However, the High Impact Practices on Social and Behavior Change for family planning presents a promising, evidence-driven framework containing best practices to support individuals and couples in reaching their family planning goals.^12^ Generally speaking, in the family planning community, the focus has switched from a procurement perspective to more rights-based and human-centered measurement approaches. Additionally, progress has been made by donors in the conceptualization and promotion of agency in the context of contraceptive use and in generating evidence on the role of agency in effective social and behavior change programming.^13^ Researchers have attempted to conceptualize contraceptive agency and argued that the agency in decision-making related to avoiding or delaying pregnancy has 6 constructs.^14^ However, interventions in another component of this composite measure—women’s ability to say no to sex—may shed some light. For example, the What Works to Prevent Violence–Impact at Scale Programme, an initiative funded by the United Kingdom, focuses on the scale-up of effective and innovative interventions to stop violence before it starts and may provide some best practices on empowering women to say no to sex.^15^

Understanding the trends and variables in women’s SRHR decision-making is crucial for developing comprehensive policies and program interventions that go beyond a sole focus on services. These interventions need to focus, in particular, on rural populations, those in the lowest wealth quintiles, and individuals with limited education, which can help bridge the significant gaps identified. It is crucial that interventions not only address directly related issues, such as violence against women or harmful practices, but also are tailored to these specific challenges and are based on a sound understanding of the specific cultural and social context to ensure that no one is left behind in achieving equitable outcomes in women’s SRHR. The diversity of trends across and within countries underscores the importance of localized, multistakeholder, data-driven approaches to enhance women’s reproductive autonomy and decision-making capabilities.

### Limitations

SDG indicator 5.6.1 and the trend analysis presented in this article are not without limitations. This indicator only measures SRHR decision-making among married or in-union women and excludes those who are not in unions, as joint decision-making dynamics typical in unions do not apply. However, SRHR is equally important among unmarried women, and different measures that are more applicable to them should be designed and tested. Another limitation of the 5.6.1 measure used in this analysis is its focus on contraceptive decision-making among users only. Recent research highlights that decision-making about non-use of contraception is also an important reflection of reproductive agency.^16^ While DHS began capturing decision-making among non-users starting with DHS7, our analysis is limited to users due to the historical availability of data for this group. We would also like to further explore the true meaning of joint decision-making, as evidence shows that women’s preferences may be undermined by men’s preferences in cases of disagreement.^17^ The majority of the data for this indicator were from low- and middle-income countries, and we lack the data needed to understand the situation of women exercising their bodily autonomy in high-income countries. Focusing only on SRHR decision-making may not fully represent women’s SRH agency. Existing literature also suggests that health care provider perspectives, partner communication and negotiation, self-efficacy, coercion and violence, social norms, and social support may be relevant.^18^^–^^26^ The present quantitative analysis was limited to the use of descriptive statistics and trend-based observations. However, the analysis in this article could further benefit from more rigorous statistical tests to verify the statistical significance of the observed changes and alternative methods of measuring inequalities, such as using the normalized concentration index for bounded variables.^27^

## CONCLUSION

As we reach the midpoint of the SDGs and celebrate the 30th anniversary of the ICPD, this is a critical moment to evaluate the progress achieved with SDG target 5.6, including indicator 5.6.1, and address the ongoing challenges in women’s SRHR decision-making. To advance sexual and reproductive agency in the SDG era and beyond 2030, policymakers and program implementers may focus on strategies, such as strengthening community-based education and outreach, particularly in rural and resource-constrained areas, that can help bridge gaps in SRHR knowledge and address the social norms that limit women’s agency. Additionally, integrating SRHR into broader policy frameworks on gender equality, violence prevention, and SRH services may create a supportive environment for women’s decision-making. Programs aimed at enhancing men’s involvement and support in SRHR while addressing barriers, such as gender and cultural norms, may further improve reproductive health outcomes. Investments in health system strengthening, including provider training, quality of care improvements, and making SRH services available, accessible, and affordable, will also be essential for creating an environment where women feel empowered to make informed decisions about their SRH. These interventions should be supported by localized, multistakeholder approaches to ensure they are data-driven, context-specific, and inclusive of the most vulnerable groups.

Looking ahead, comprehensive global data coverage will be necessary to ensure that the evidence gathered influences policy and program decisions at the global, regional, national, and subnational levels. Effective action on these fronts will help lay a foundation for achieving equitable SRHR outcomes and ensure that no one is left behind in achieving women’s sexual and reproductive autonomy.

## Notes

**Peer Reviewed**
